# Effects of bone surface topography and chemistry on macrophage polarization

**DOI:** 10.1038/s41598-024-62484-3

**Published:** 2024-06-03

**Authors:** Birgün Özcolak, Berkay Erenay, Sedat Odabaş, Klaus D. Jandt, Bora Garipcan

**Affiliations:** 1 Biomimetic and Bioinspired Biomaterials Research Laboratory, Institute of Biomedical Engineering, Boğaziçi University, 34684 Istanbul, Turkey; 2 Department of Biomedical Engineering, School of Engineering and Natural Sciences, Istanbul Medipol University, 34810 Istanbul, Turkey; 3 Biomaterials and Tissue Engineering Laboratory (bteLAB), Department of Chemistry, Faculty of Science, Ankara University, 06560 Ankara, Turkey; 4 Interdisciplinary Research Unit for Advanced Materials (INTRAM), Ankara University, 06560 Ankara, Turkey; 5 Chair of Materials Science (CMS), Otto Schott Institute of Materials Research, Faculty of Physics and Astronomy, Friedrich Schiller University Jena, Löbdergraben 32, 07743 Jena, Germany

**Keywords:** Biomimetic, Bone surface topography, Soft lithography, Surface modification osteoimmunomodulation, Macrophages, Macrophage polarization, Biomaterials - cells, Biomimetics

## Abstract

Surface structure plays a crucial role in determining cell behavior on biomaterials, influencing cell adhesion, proliferation, differentiation, as well as immune cells and macrophage polarization. While grooves and ridges stimulate M2 polarization and pits and bumps promote M1 polarization, these structures do not accurately mimic the real bone surface. Consequently, the impact of mimicking bone surface topography on macrophage polarization remains unknown. Understanding the synergistic sequential roles of M1 and M2 macrophages in osteoimmunomodulation is crucial for effective bone tissue engineering. Thus, exploring the impact of bone surface microstructure mimicking biomaterials on macrophage polarization is critical. In this study, we aimed to sequentially activate M1 and M2 macrophages using Poly-l-Lactic acid (PLA) membranes with bone surface topographical features mimicked through the soft lithography technique. To mimic the bone surface topography, a bovine femur was used as a model surface, and the membranes were further modified with collagen type-I and hydroxyapatite to mimic the bone surface microenvironment. To determine the effect of these biomaterials on macrophage polarization, we conducted experimental analysis that contained estimating cytokine release profiles and characterizing cell morphology. Our results demonstrated the potential of the hydroxyapatite-deposited bone surface-mimicked PLA membranes to trigger sequential and synergistic M1 and M2 macrophage polarizations, suggesting their ability to achieve osteoimmunomodulatory macrophage polarization for bone tissue engineering applications. Although further experimental studies are required to completely investigate the osteoimmunomodulatory effects of these biomaterials, our results provide valuable insights into the potential advantages of biomaterials that mimic the complex microenvironment of bone surfaces.

## Introduction

Surface topography is one of the crucial factors that affect the cellular response to biomaterials such as adhesion, differentiation, and proliferation^1^. Recent studies have shown that different surface topographies can also impact the immune cell response, including macrophage polarization^2–4^. This has led to a growing interest in the field of osteoimmunomodulation, which seeks to understand how the immune system responds to various stimuli and how these responses affect immune function and bone health. Traditionally, osteoblastic cells have been used to evaluate the in vitro efficacy of biomaterials in bone tissue engineering applications. However, the role of the immune response in tissue repair has not been fully considered in conventional approaches^5–7^.

During wound healing, macrophages assume two distinct phenotypes, M1 and M2, which are important for different stages in the process^7–9^. M1 macrophages are present immediately after tissue injury (1–3 days) as they help to control the early inflammatory phase^10^. Additionally, M1 macrophages play a crucial role in initiating angiogenesis and osteogenesis. After the initial 3 days, M1 macrophages are gradually replaced by M2 macrophages, which serve to reduce inflammation and stimulate new tissue formation^10^. Injuries with suboptimal healing outcomes often result from a disruption in the transition of macrophages from the M1 to M2 phenotype. To enhance the healing process, a proposed strategy involves biomaterials that promote the sequential activation of M1 followed by M2 macrophages. This approach is considered more effective in promoting healing compared to activating either phenotype in isolation or concurrently promoting both. A swift transition from M1 to M2 activation accelerates the recovery process. It is well-established that M1 and M2 macrophages play synergistic and sequential roles in tissue repair^5–7,9^. To improve tissue repair outcomes, it is necessary to explore the impact of surface topography on immune cell behavior and develop biomaterials that can modulate the immune response^5–7^. There are six possible strategies for constructing biomaterials-mediated immunomodulatory responses; adding immuno-modulatory drugs onto or into biomaterials^11,12^, varying the materials’ particles size^13–16^, adjusting materials’ pore size^17^, altering the surface wetting properties of the materials^18^, combining biomaterials with bioactive elements like Ca, Mg, Si, Sr^19,20^, and changing the surface topography of materials^21,22^. Combining surface topography with immune responses presents an innovative approach in biomaterials design, emphasizing the importance of modifying surface features to influence biomaterials’ osteoimmunomodulative properties.

Bone has an anisotropic surface structureand surface topography is a potential modulator of the immune response^23,24^. Wang et al*.* found that 1D wrinkles that generate grooves and ridges have been shown to promote M2 polarization; whereas, 2D wrinkles that create pits and bumps, can stimulate M1 polarization^25^. Chen et al*.* suggested that surface topography can have an impact on macrophage behavior regardless of surface chemistry. Specifically, the increase in topography feature size from 500 nm to 2 μm can restrict macrophage activation and inflammation-related activities^26^. Barth et al*.* also found that macrophages seeded onto rougher surfaces tend to exhibit an M2-like phenotype, which suppresses immune and anti-inflammatory processes, plus promotes angiogenesis, extracellular matrix formation, and tissue repair^27^. Similarly, Damanik et al*.* report that microporous structures trigger macrophages to exhibit an M2-like phenotype^28^. It is important to note that while previous studies have focused on producing the rough and porous characteristics resembling bone surfaces, none has yet attempted to mimic the natural surface topography of the bone. This gap underlines the untapped potential of investigating how these unique surface features can be mimicked to better guide the immune response for desired outcomes.

Regarding the materials perspective, natural bone is a complex composite, primarily made up of collagen and calcium phosphate (CaP) in the form of nanocrystalline hydroxyapatite (HA)^29,30^. Collagen nanofibrils make up the main component of the organic matrix, providing natural bone with excellent tensile strength and toughness^30,31^. Meanwhile, HA nanocrystals, the inorganic phase, provide natural bone with high stiffness and compressive strength, acting as a natural reservoir of calcium, phosphate, and other inorganic ions^30,32^. Over the past 40 years, researchers have used biomimetic approaches to develop synthetic bone graft substitutes by synthesizing and fabricating composites made up of various polymers, collagens, and CaP phases^30^. Laquerriere et al*.* suggested that small-diameter hydroxyapatite particles can activate immune cells to release pivotal cytokines, which favor inflammation^15^. Fernandes et al*.* reported that hydroxyapatite granules activate M2 polarization of THP-1 cells more than M1^33^. According to Sadowska et al*.,* hydroxyapatite-coated materials play an important role in triggering the formation of pro-inflammatory cytokines and controlling osteogenic processes^34^. Li Jiashen et al*.* reported that Col-I modified membranes do not have cytotoxic effects on adipose-derived stem cells and can initiate osteogenic differentiation of these cells^35^. Moreover, Col-I can affect the expression of osteogenic factors such as WNT10b, BMP2, BMP6, and OSM, as it generates a rough surface on the material^36–38^. Correia et al. found that unmodified PLA activates M1 macrophage polarization, while Col-I modified PLA macrophages with the same surface properties generally prompt M2 macrophage polarization^39^. Although there are numerous studies characterizing the osteogenic properties of materials comprising CaP and type 1 collagen (Col-I), knowledge about their osteoimmunomodulatory properties is limited in the literature^40–43^. Investigating deeper into the osteoimmunomodulatory aspects of CaP and Col-I-based materials can potentially elucidate strategies for designing materials that actively mimic the tissue microenvironment for desired immune responses.

Reports from current literature have shown that the surface topography and chemistry of biomaterials significantly affect macrophage behavior. Bone-like rough and microporous surfaces have been suggested to promote tissue repair and angiogenesis while suppressing immune and anti-inflammatory responses. However, none of the different surface topographies used in the literature directly mimic the natural surface topography of bone. To address this critical gap, our hypothesis is that the fabrication of a bone-like anisotropic microenvironment by mimicking bone surface topography and chemistry can lead to a desired osteoimmunomodulatory response. To this end, our research endeavors are diligently focused on establishing a foundational concept and gaining an in-depth understanding of the interaction between biomaterials and the polarization of RAW cells. For our macrophage model, RAW264.7 cells were chosen due to their well-established characteristics, ease of cultivation, and practicality for experimentation. Their high proliferation rate simplifies various assays and ensures experimental consistency^44,45^. Moreover, Raw264.7 cells offer a relevant choice for predicting our biomaterials' effects on human cells, reflecting potential human reactions and widely employed in various studies^46–50^. Our primary investigative emphasis has been on examining key parameters such as cell viability, morphology, and cytokine release, with the ultimate goal of providing a proof of concept. However, it is paramount to optimize this methodology and embark on more comprehensive research to enhance the accuracy of M1/M2 phenotype classification. In previous studies, our research group utilized soft lithography to fabricate bone surface topography-mimicked (BSM) membranes, employing a bovine femur as a representative model surface. Puza et al. highlighted that BSM chitosan membranes promote enhanced cell viability and improved cell morphology in osteoblast cells. Similarly, Erenay et al. demonstrated that BSM PDMS membranes elicit an increase in cell proliferation, attachment, and the expression of markers linked to the differentiation and maturation of osteoblast cells^51,52^. The focus of the current study is to assess the osteoimmunomodulatory properties of BSM membranes using FDA-approved biodegradable Poly-L lactic Acid (PLA)^53^, as a model polymer. Moreover, BSM membranes were modified with Col-I and HA to emulate bone surface microstructure. Subsequently, RAW 264.7 cells were cultured on these prepared BSM membranes to evaluate their osteoimmunomodulatory characteristics by analyzing cell viability and determining the dominant macrophage phenotype by using ELISA and SEM analysis. It was observed that hydroxyapatite deposited BSM membranes showed sequential M1 and M2 polarizations, suggesting potential for bone tissue engineering applications by modulating the immune response. While additional experimental investigations are required to fully explore the osteoimmunomodulatory effects of bone surfaces mimicked biomaterials. Additionally, our findings offer significant perspectives on the potential benefits of biomaterials that mimic the complex microenvironment of bone surfaces. Our innovative approach seeks to overcome previous limitations and design biomaterials that effectively guide immune responses for improved bone tissue engineering outcomes, mimicking bone's unique surface topography and microstructure.

## Materials and methods

### BSM membrane production

#### Preparation of BSM membranes

##### Bone surface cleaning

Bovine femur bones were selected as the foundational templates for the creation of BSM membranes and were supplied from a local butcher. To ensure the bone samples were suitable for this purpose, a rigorous xenograft cleaning process was undertaken. Specifically, only the outer surface of the diaphysis portion of the cortical bone, located at least 10 cm away from any cutting points, was chosen to yield bone pieces measuring 2 cm by 2 cm.

The cleaning process involved a series of meticulous steps. Initially, the bone pieces were immersed in absolute ethanol for 30 min to cleanse the surface thoroughly. Subsequently, they were transferred into a 10% sodium chloride (NaCl) solution for a duration of 24 h. Notably, during this transfer, the bone pieces underwent ultrasonic bath treatment during the initial and final 20 min, ensuring the complete removal of any residual cells and tissues from the bone surface.

Following the NaCl treatment, the bone pieces were immersed in acetone for 20 min to eliminate lipids and any other potential surface residues. To address immunologic residues and inactivate prions, the samples were subjected to a sequence of solutions: 3% hydrogen peroxide (H_2_O_2_) for 72 h, 2 M sodium hydroxide (NaOH) for 2 h, and acetone for an extended period of 144 h.

Subsequently, after the chemical cleaning process, the bone specimens were allowed to air-dry naturally at room temperature, ensuring the complete removal of any chemical residues. These meticulously cleaned bone pieces were then ready for incorporation into the subsequent stages of the soft lithography process, facilitating the creation of BSM membranes with a high degree of precision and biocompatibility^51,52^.

##### Preparation of PDMS molds

Bone surface topography was mimicked by using soft lithography technique. Polydimethylsiloxane (PDMS; Sylgard 184, Dow Corning, USA) solution was prepared with a prepolymer cross linker ratio of 10:1 (w/w). The prepared PDMS solution was degassed in vacuum chamber for 30 min and poured onto the pre-treated bone and cured at 70 °C for 4 h.

##### Production of BSM PLA membranes

BSM negative PDMS molds were used to fabricate BSM membranes by using PLA polymer. PLA (10% (w/v), intrinsic viscosity, 1.8 dL/g, 221.000 g/moL MW) was dissolved in chloroform (Sigma Aldrich-24216) and cast onto BSM negative PDMS molds for 12 h. As a negative control, plain PLA membranes were also fabricated using plain PDMS molds.

### Col-I and HA Immobilization of the BSM PLA Membranes

To mimic bone microstructure biochemically, BSM PLA membranes were modified with HA and Col-I after being activated with O_2_ plasma treatment at 100 W Power, 80 Pa Pressure for 10 min. For Col-I modification, membranes were incubated with 1-Ethyl-3-[3-dimethylaminopropyl] carbodiimide hydrochloride (EDAC; E1769, Sigma-Aldrich, USA) phosphate buffer solution (10 mg/mL, pH 7.4) for 4 h at 0 °C to activate the COOH residues on the surface of the membranes. Finally, these membranes were transferred into the phosphate buffer solution (pH 4.5), containing 0.05, 0.1, 0.25, 0.5 mg/mL Col-I amount for 24 h, and membranes were kept in deionized water at 37 °C for 24 h to remove unbound collagen from the surface^54^. For HA modification, the membranes were immersed into 1% (w/v) HA in ethanol suspension at room temperature for 1 h under stirring after O_2_ plasma treatment^55^. Plain PLA membranes were modified using the same procedure.

### Optimization of Col-I immobilization

#### Immunohistochemical analysis for collagen immobilized area calculation

The maximum surface coverage with optimum Col-I amount was analyzed by immunohistochemical staining, which was performed for unmodified, and Col-I modified BSM PLA membranes. For blocking, the membranes were incubated into 10% normal goat serum in PBST (PBS + 0.1% Tween 20) for 30 min. Blocked membranes were incubated in 1:100 diluted primary antibody (SC-59772, Santa Cruz, CA, USA) overnight at 0 °C and then membranes were incubated in 1:200 diluted secondary antibody [ab97057, Goat Anti-Rat IgG H&L (HRP)] for 10 min at room temperature. After incubation in 3,3-diaminobenzidine tetrahydrochloride substrate chromogen solution (Cat. No. 34002, Pierce™ DAB Substrate Kit, Thermo Scientific) for 5 min, Col-I modified BSM PLA membranes were stained. The images of the stained membranes were taken by using an digital microscope (HIROX KH-8700)^56^. Col-I coating area percentage was calculated by using Image J software by pixel counting.

#### Immobilized Col-I concentration analysis via sirius red staining

The total collagen content on the collagen immobilized biomaterials was measured by Sirius Red/Fast Green Collagen Staining Kit (9046, Chondrex) according to the manufacturer’s instructions. This technique was applied to examine both unmodified and Col-I modified plain, as well as BSM PLA membranes. To ensure reliability, all experiments were conducted in triplicates. The unmodified plain PLA membranes were utilized as the designated control group for comparative analysis.

### Characterization of the Col-I and HA modifications

#### Wettability analysis

Sessile drop water contact angle analysis was used to assess wettability properties of unmodified, HA coated, and Col-I modified plain and BSM PLA membranes (CAM 100, KSV, Finland). Deionized water droplets (10 µL) were dropped onto surfaces, and water contact angles were measured by circle fitting. All measurements were performed at room temperature. All water contact angle measurements reported in this study are the average of 8 different measurements per experimental group and they were taken from 2 different regions within each experimental group.

#### Fourier transform infrared spectroscopy (FTIR)

FTIR spectra were recorded over the range of 4000–400 cm^−1^ with 32 scans to examine the surface chemical compositions of the unmodified, HA-coated and Col-I modified PLA membranes. The FTIR measurements were performed by using Attenuated Total Reflectance Fourier Transform (ATR- FTIR) spectrophotometer (Perkin Elmer, Spectrum 100, USA)^57^.

#### X-ray photoelectron spectroscopy (XPS)

To determine the compositions of unmodified and Col-I modified membranes, X-ray photoelectron spectrophotometry (X-ray Photoelectron Spectrometer, Thermo Scientific K-Alpha, UK) was utilized with an Al Kα radiation (photon energy 1000 eV) excitation source. Survey spectra were taken over the range of 1350 to 0 eV with a pass energy of 150 eV^58^.

#### X-ray diffraction (XRD)

The phase composition of unmodified and HA modified membranes was characterized by X-ray diffraction (XRD, D/max-2500X, Japan) with monochromated Cu Kα radiation (λ 1⁄4 1.5405 Å, 120 mA, 40 kV) in a continuous scan mode with 81/min scanning speed as well as the 2θ range was from 10 to 70^59^.

### Characterization of the surface topography of the BSM membranes

#### Atomic force microscopy (AFM)

Sample surface topographies of the HA or Col-I modified and unmodified Plain PLA and BSM PLA membranes (diameter 15.6 mm) were analyzed via AFM (Park Systems Park Systems, Korea) equipped with NSC36B silicon cantilever in non-contact mode. Scan fields of 12 × 12 µm^2^ on all surfaces were processed using the XEI software (1.18).

#### Scanning electron microscopy (SEM)

To observe bone surface mimicking quality and the possible alteration in surface topography after Col-I or HA modifications, surface topography of modified and unmodified Plain PLA and BSM PLA membranes were examined by SEM (EVO 40 Series, Carl Zeiss AG, Germany). Observed areas were pinpointed by creating a reference point on the bone piece prior to preparation of the PDMS negative mold. All surfaces were coated with thin layer of platinum (50 nm).

### Cell culture studies

**Figure 1:**
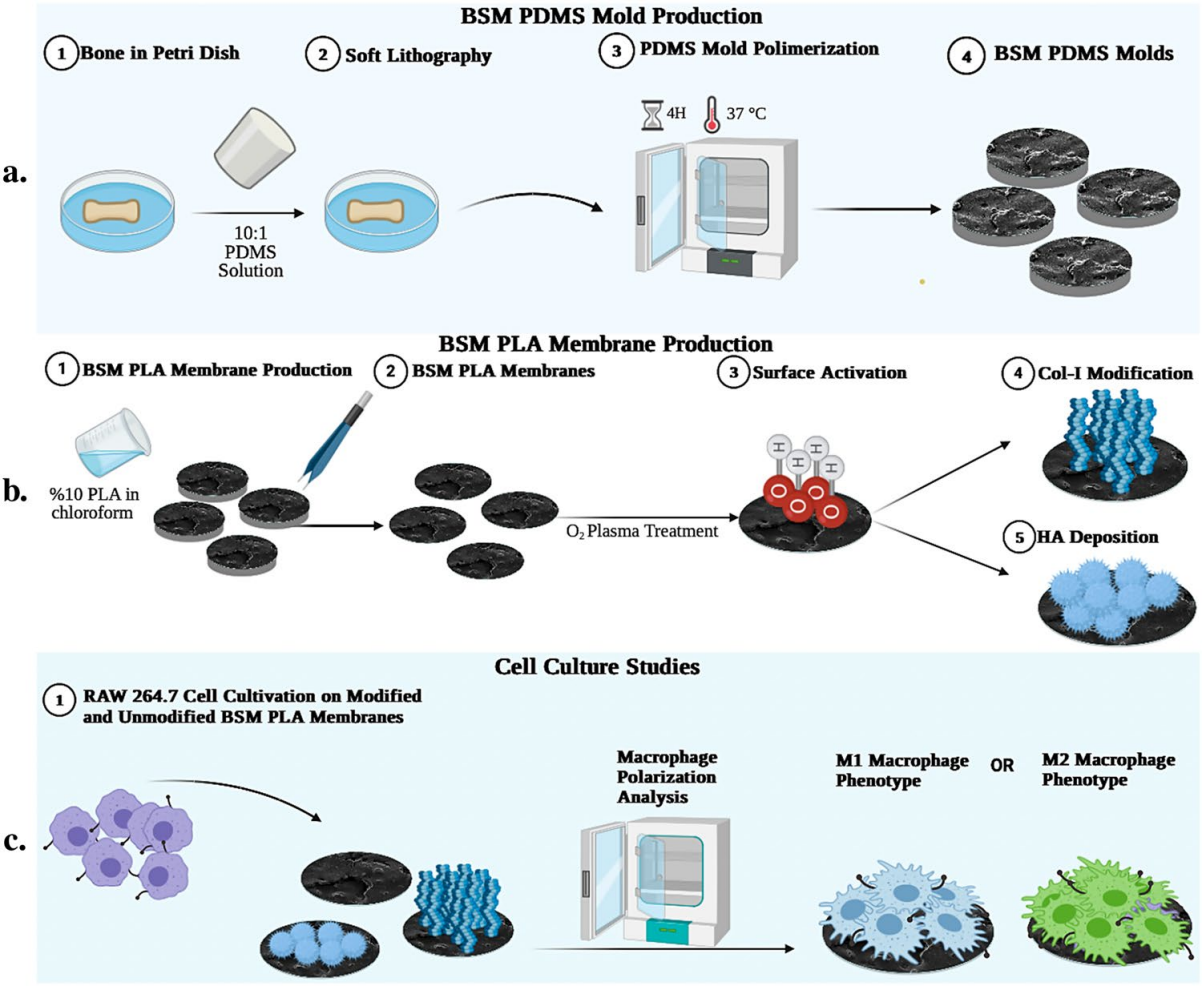
Schematic of BSM membrane production. (**a**) BSM-PDMS Molds preparation process. Negative molds were initially created using bone samples, and these molds were subsequently utilized to fabricate BSM-PLA membranes. (**b**) Unmodified and Modified BSM-PLA membrane production process. (**c**) RAW 264.7 cell culture studies. RAW 264.7 cells were cultured on unmodified, and Col-I and HA modified plain and BSM membranes to investigate macrophage phenotypes using SEM and ELISA.

RAW 264.7 cell line was purchased from American Type Culture Collection (ATCC^®^ TIB-71™). Cells were cultured in high glucose Dulbecco's Modified Eagle’s Medium (DMEM; D5796, Sigma-Aldrich) supplemented with 10% fetal bovine serum (FBS; F9665, Sigma-Aldrich) and 1% penicillin/streptomycin, in an atmosphere of 5% CO2 and 95% humidity at 37 °C. Cells were passaged after reaching 80% confluence, detached with cell scraper, and were passaged in 1:6 ratio in T-75 flasks. Cells were cultured until passage number: 5. RAW 264.7 (4 × 10^4^) cells were seeded on modified and unmodified plain PLA and BSM PLA membranes (Fig. 1) consisting of DMEM high glucose (D5796, Sigma Aldrich), 10% FBS (F9665, Sigma-Aldrich), and 1% Penicillin/streptomycin (P4333, Sigma-Aldrich).

#### Cell viability

The effect of plain and BSM modified and unmodified membranes on viability of the RAW 264.7 cells analyzed via WST-1 assay by using a commercial kit according to the manufacturer’s instructions (ab65473; Abcam, Cambridge, MA) at day 1, day 3, and day 6. Experiments were conducted in triplicates and plain PLA membranes were accepted as a control group.

#### Cell morphology analysis

After incubation, the RAW 264.7 cells were fixed with 2.5% glutaraldehyde (Sigma-Aldrich, G5882). Then, fixed cells were dehydrated by incubating them in graded ethanol series (30%, 50%, 70%, 90% and 100%). Finally, the cells were dried with a hexamethyldisilazane (HMDS, 440191, Sigma-Aldrich) solution for 5 min^60,61^. Before analysis, the samples were sputter coated with a thin layer of gold (Au) and observed under SEM (Carl Zeiss EVO 40, Germany) at 10 kV.

#### Cytokine content analysis in the supernatant

To determine the dominant macrophage phenotype in osteoimmunomodulation regulation, we utilized double antibody sandwich ELISA kits for colorimetrical assessment of the ratio of proinflammatory and antiinflammatory factors of monocytes/macrophages. RAW 264.7 cells were incubated on unmodified and modified, plain PLA and BSM PLA membranes for 1 day, 3 days, and 6 days, and then the media were collected. Supernatants were obtained from the collected medium by centrifugation at 210 g and stored at − 80 °C until use. The presence and amount of proinflammatory (IL-6 and IL-1β) and antiinflammatory (IL-1ra and IL-10) factors in the stored supernatants were analyzed using appropriate ELISA kits (R&D Systems, Gymea, NSW, Australia), according to the manufacturer's instructions. A calibration curve was obtained for each cytokine to determine unknown concentrations. Calibration curves are provided in the supporting information Fig. S1. Experiments were conducted in triplicates^62,63^.

### Statistical analysis

Results were given as mean ± standard deviation and differences were considered as statistically significant for *p* < 0.05. GraphPad Prism 9 (GraphPad Software, USA) was used to perform the statistical analyses. The data were analyzed using two-way ANOVA followed by Tukey’s post hoc multiple comparison test. Plain PLA was accepted as a control group for viability and cytokine release analyses, and Plain Col-I 0.05 was accepted as a control group for Immunohistochemistry and Sirius Red analyses.

## Results and discussion

### Characterization of the Col-I and HA modifications

#### Optimization of collagen immobilization

**Figure 2:**
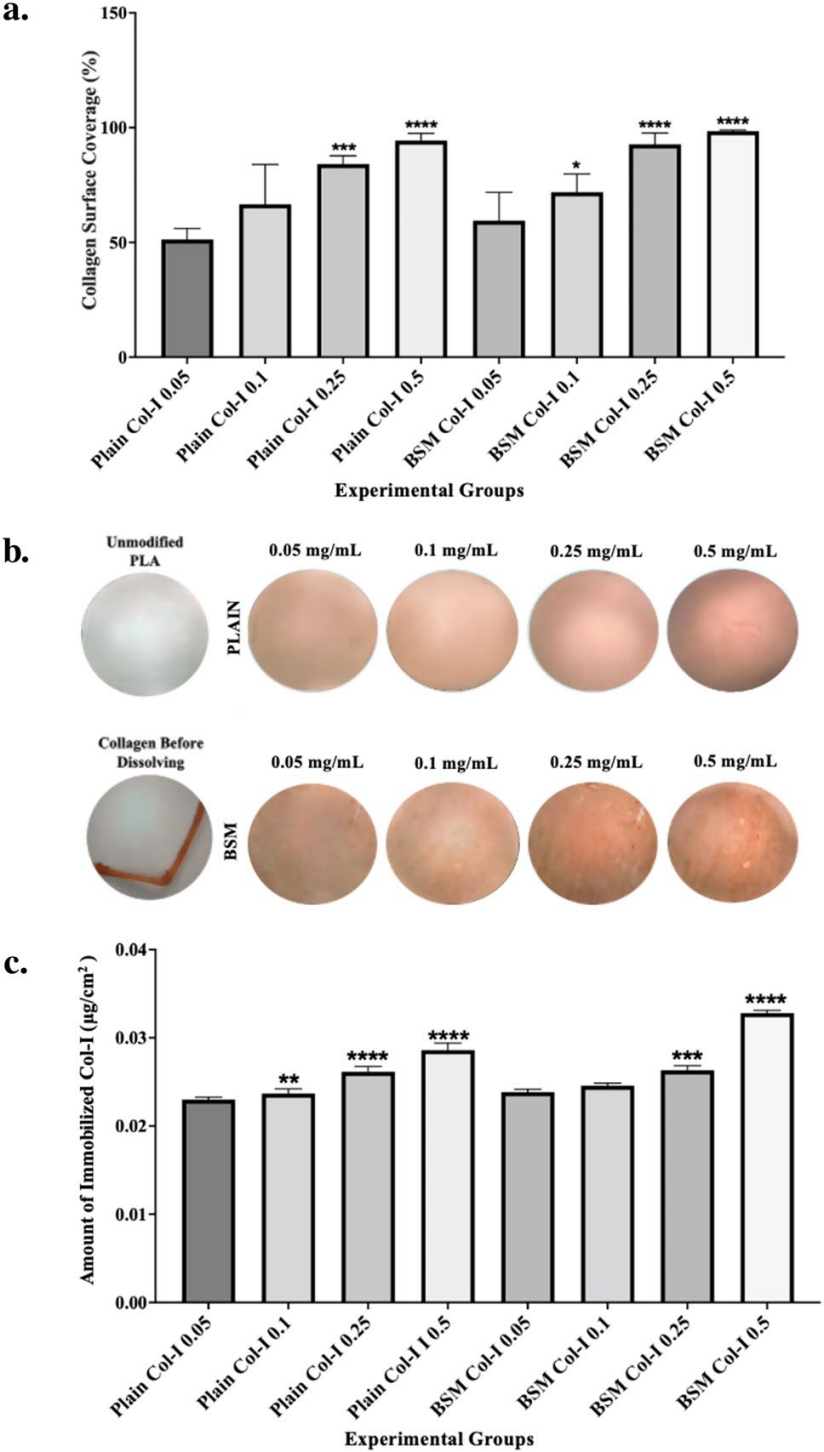
(**a**) Immunohistostaining analysis of unmodified and Col-I modified plain and BSM PLA membranes by using Image J, (**b**) Immunohistostaining of unmodified and Col-I modified plain and BSM PLA membranes, (**c**) Quantification of Col-I content by Sirius Red assay (*p < 0.05, **p < 0.01, ***p < 0.001, ****p < 0.0001).

Immunohistochemistry analysis was conducted to determine the amount of Col-I immobilization, and microscopy images of both unmodified and modified PLA were taken by using digital microscopy and analyzed by using ImageJ software. The results showed that an increase in Col-I concentration in the immobilization solution led to an increase in Col-I coating area percentage. As depicted in Fig. 2a,b, 0.05 mg/mL of Col-I concentration covered 51.26 ± 4.22% of the Plain PLA membrane surfaces and 59.43 ± 10.67% of the BSM PLA membrane surfaces. When the Col-I concentration was increased to 0.1 mg/mL, the coating area percentage increased to 66.63 ± 14.96% for Plain PLA membrane surfaces and 71.84 ± 6.88% for BSM PLA membrane surfaces. A concentration of 0.25 mg/mL resulted in a coating area percentage of 84.10 ± 3.15% for Plain PLA membrane surfaces and 92.73 ± 4.31% for BSM PLA membrane surfaces. The highest Col-I coating area percentage was achieved with a concentration of 0.5 mg/mL, which coated 94.36 ± 2.75% of the Plain PLA membrane surface and 98.43 ± 0.43% of the BSM PLA membrane surface.

The maximum immobilized amount of Col-I for each experimental group (0.05 mg/mL, 0.1 mg/mL 0.25 mg/mL and 0.5 mg/mL) was analyzed by using Sirius Red staining and given at Fig. 2c. 0.353 ± 0.007 µg/cm^2^ of Col-I was immobilized onto BSM membranes after having been incubated with 0.5 mg/mL Col-I solution, and it resulted in the highest immobilized Col-I amount and was significantly statistically different compared to other experimental groups. Then, 0.241 ± 0.017 µg/cm^2^ Col-I was immobilized onto Plain membranes which were incubated in 0.5 mg/mL Col-I solution, and it resulted in being the second highest Col-I amount and significantly statistically different from each experimental group. Therefore, 0.5 mg/mL Col-I concentration was found the optimum concentration for the immobilization Col-I according to immobilized protein amount analysis.

#### Wettability analysis

**Figure 3:**
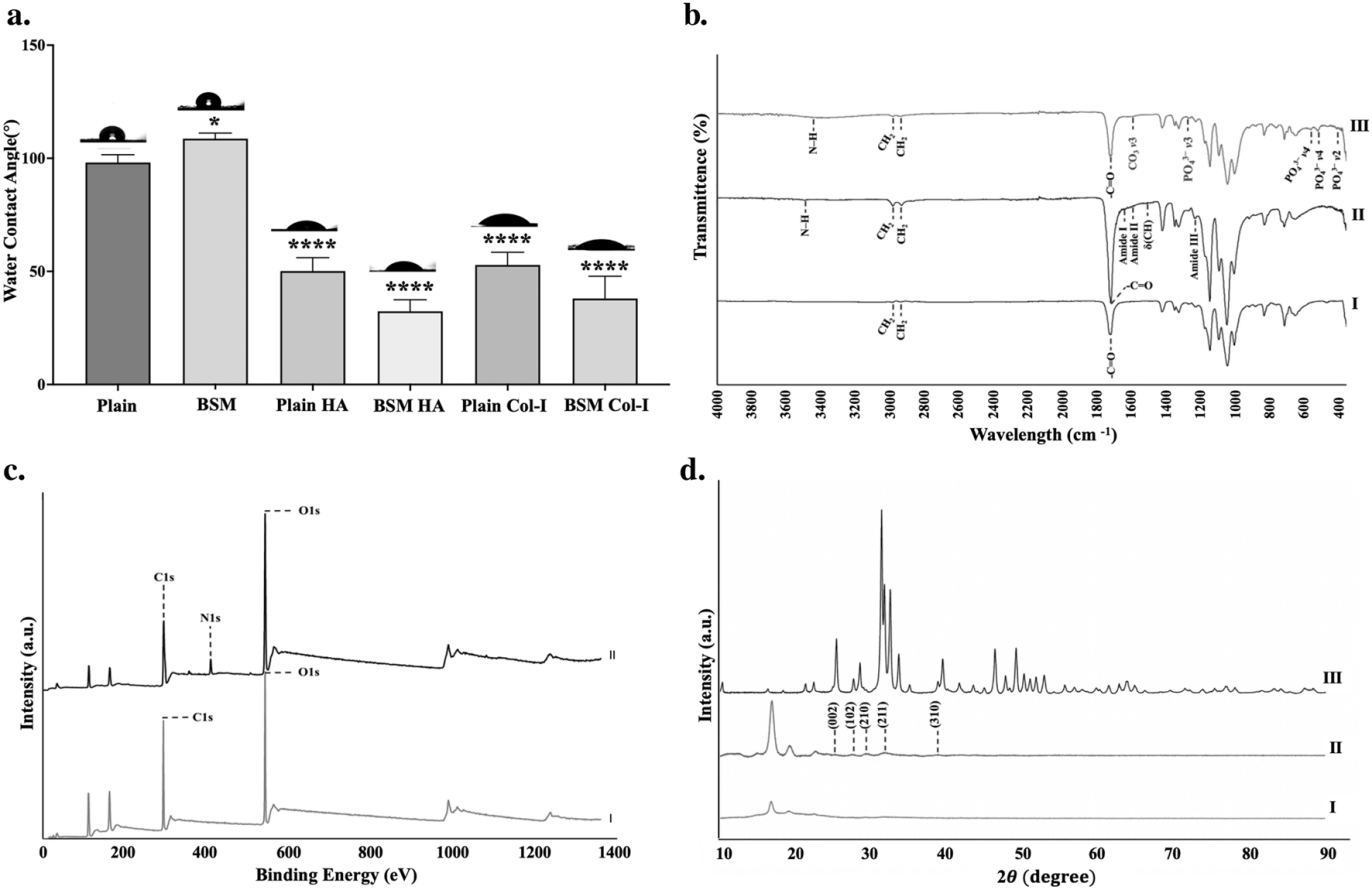
Characterization of surface wettability, chemical composition, and crystallinity of membranes. (**a**) WCA analysis, (**b**) FTIR analysis; I. HA modified PLA, II. Col-I modified PLA, III. Unmodified PLA (**c**) XPS analysis; I. Col-I modified PLA, II. Unmodified PLA, (**d**) XRD Analysis I. Unmodified PLA, II. HA modified PLA, III. Pure HA.

Biomaterial surface wettability is regulated mainly by chemical properties and surface topography^64^. Hydrophobicity/hydrophilicity affect the protein adsorption behavior on the surface, which in turn influences cell adhesion and proliferation^64^. To estimate the effect of the surface topography and the surface chemical modifications on the surface wettability, water contact angle values (WCA) were measured (Fig. 3a). WCA values of plain PLA and BSM membranes with and without HA and Col-I modified are given in Fig. 1. Contact angle value of plain PLA was measured at 108.67° ± 2.2° which was similar to the values reported for PLA surfaces by Schaub et al*.*^65^. Ma et al*.* suggested that after collagen type-I was immobilized on the BSM PLA surface, the WCA reduced from 71.0° ± 1.6° to 35.0° ± 3.4°. Contact angle value of Col-I modified PLA was 52.8° ± 5.3°. Thereby, the hydrophilic property of the Col-I modified samples was rised two-fold which was parallel to the results of Ma et al.^66^. WCA measurement of the unmodified plain PLA was 98.1° ± 3.2°. The WCA result of the HA modified plain PLA was 50.1° ± 5.6°. According to Ge et al*.* the WCA measurements of the HA coated PLA membranes dropped from 131.9° to 70.3°, which was in agreement with the obtained results^67^. In addition, unmodified BSM PLA membranes were more hydrophobic than plain PLA membranes (98.1° ± 3.2° and 108.7° ± 2.3°, respectively). Col-I and HA modified BSM PLA membranes whose WCA values were measured as 38.0° ± 9.2° and 32.4° ± 4.8°, respectively, compared to their plain counterparts, whose WCA values were taken as 52.8° ± 5.3° and 50.1° ± 5.6°, respectively. These results were in line with the Wenzel Theorem^68^, which explains the relation of surface hydrophilicity with material surface roughness. Increased surface roughness on a material will make hydrophobic materials turn into more hydrophobic and hydrophilic materials turn into more hydrophilic^68^.

#### Surface chemistry analysis

The composition of Col-I and HA modified PLA membranes was analyzed by FTIR analysis. Col-I modified membranes showed Amide A, Amide I, Amide II and Amide III peaks in their FTIR spectra (Fig. 3b). The peak centered at 3350 cm^−1^ involves a mutual band of hydrogen bonds from the intermolecular water and amide A of collagen related to N–H stretching. The peak around 1650 cm^−1^ was accepted as an amide I band and originated from C=O stretching vibrations coupled with N–H bending vibrations^69^. The peaks around 1545 cm^−1^ and 1238 cm^−1^ were accepted as amide II and amide III bands, respectively^69^. It can be noted that the peak around 1379 cm^−1^ was accepted as an δ(CH2) band. Thereby, Col-I modification on the PLA membranes was confirmed with this data^69^. In IR spectra of the HA deposited membranes, characteristic bands were observed. Peaks observed around 485 cm^-1^, 1200 cm^−1^, 524 cm^−1^, and 627 cm^−1^ were corresponded to a _*V2*_ PO_4_^3−^, _*V3*_ PO_4_^3−^ and _*V1*_ PO_4_, respectively. HA deposited membranes also exhibited a characteristic carbonate band. The n3 mode of CO_3_^2−^ at 1355 cm^−1^ was covered by a broad band because of the occurrence of CO_3_^2−^^70,71^. The presence of specific characteristic peaks and bands in the FTIR spectra provided compelling evidence for the successful modifications.

The surface chemical compositions of unmodified and Col-I modified PLA membranes were evaluated by XPS analysis and the results were given in Fig. 3c. XPS spectra demonstrated that the C1s and O1s peaks of unmodified and Col-I modified PLA membranes were alike to each other. The C1s spectra showed peaks at 284.8 eV, 287.1 eV, and 289.3 eV which related to C–C or C–H, C–O, –CO–O respectively^72^^,^^73^. O1s peak was adjusted with a single component at 531.8 eV corresponding to O=C–N interactions^74^. Furthermore, although there were not any N1s peaks in the XPS analysis result of the unmodified PLA membranes, Col-I modified PLA membranes had characteristic N1s peak at 410 eV^75^. The results of the XPS analysis provided complementary evidence to the FTIR data, demonstrating the success of the modifications, and enlightening the chemical changes taking place on the surface of the PLA membranes.

To prove the deposition of the HA on the modified membrane surfaces, pure HA powder, HA modified PLA membranes, and unmodified PLA membranes, static XRD analysis was performed. The results were shown in Fig. 3d. Peaks seen in the XRD patterns of HA modified PLA membranes and HA powder can be assigned to the (002), (102), (210), (211), and (310) crystallographic planes of hydroxyapatite^76^. Thereby, the XRD analysis provided valuable insight, underscoring the clear evidence of hydroxyapatite-associated peaks in both the HA powder and the HA-coated PLA membranes. In striking contrast, these distinctive peaks were conspicuously absent in the XRD patterns of the unmodified PLA membranes. This compelling observation serves as robust evidence, strengthening the claim that HA was successfully anchored onto the PLA membrane surfaces.

### Characterization of the surface topography of the unmodified and modified BSM membranes

#### Scanning electron microscopy (SEM)

**Figure 4:**
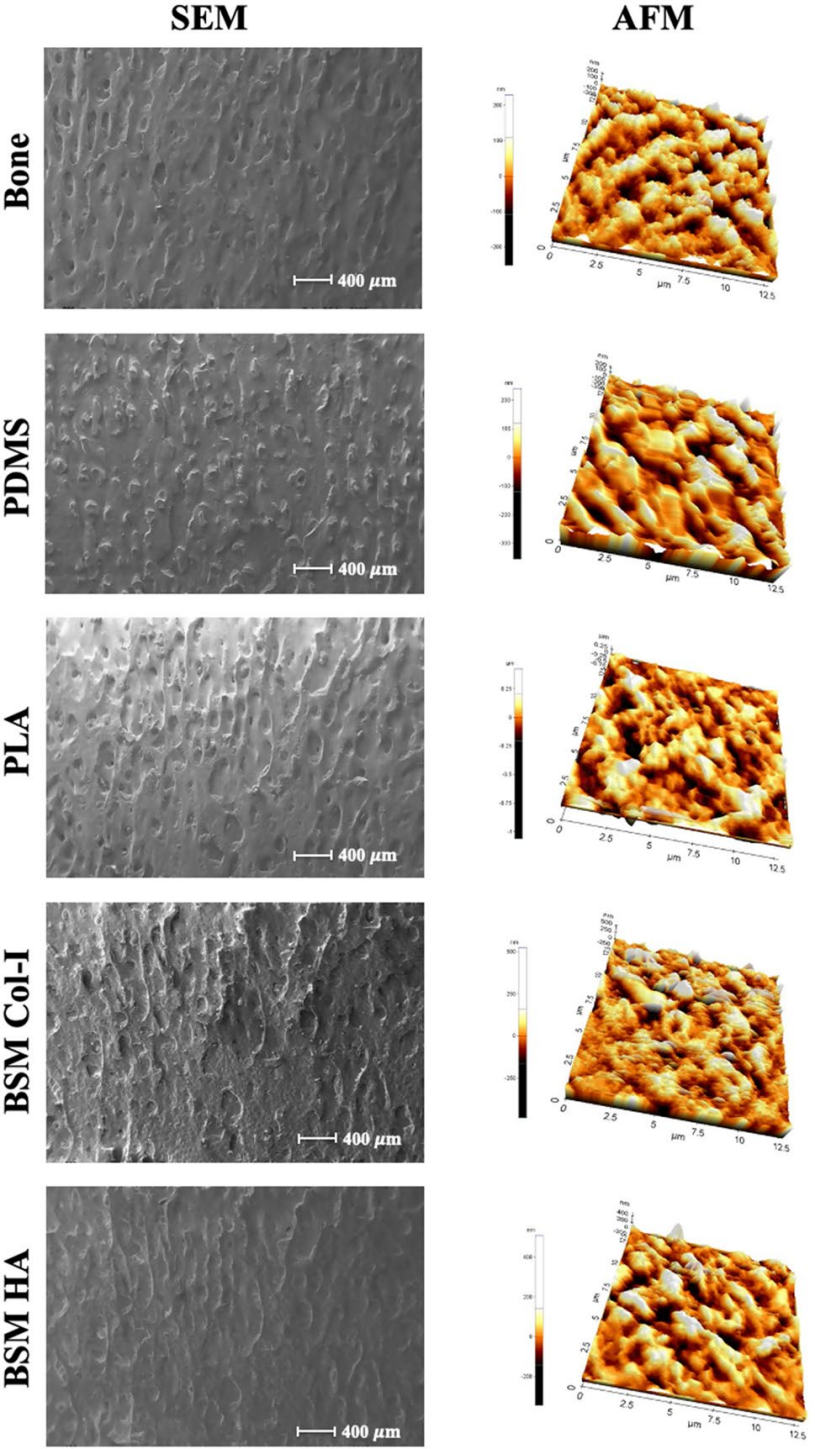
SEM and AFM images of the surface 3-D structure of the bone, BSM PDMS mold, unmodified, Col-I and HA modified plain and BSM PLA membranes.

The quality of mimicking was examined by comparing the SEM images from the same areas on the bone piece, its PDMS negative mold, and its corresponding unmodified and modified BSM PLA membranes. These areas were compared to each other by creating a reference point by making a dent on the bone piece prior to preparation of the PDMS negative mold*.* The natural bone surface topography comprises microstructures with diverse heights and pits. The surface topography of all protrusions and intrusions of the bone was completely mimicked both onto the PDMS negative mold and, from there, onto the PLA membranes (Fig. 4). Based on the findings, it can be inferred that the utilization of soft lithography (molding) and solvent casting of PLA offers an effective approach for mimicking bone surface topography, encompassing anisotropic microstructures. After Col-I and HA modifications, the varied heights and pits of the bone surface can still be observed on the BSM membranes produced using the soft lithography technique. Therefore, it can be said that the modifications did not disrupt the mimicked bone surface microstructure.

#### Atomic force microscopy (AFM)

To investigate the surface features of the bone, BSM PLA, HA modified, and Col-I modified BSM PLA membranes, non-contact AFM analysis was performed on the samples. As shown in Fig. 4, the surface features of the bone were a mirror image of the PDMS mold, while the unmodified and Col-I and HA modified BSM PLA surface features were like the bone itself.

The roughness values of the bone and BSM PDMS molds were calculated to be 50.02 nm and 56.36 nm, respectively. The roughness value of the BSM PLA membrane was calculated to be Ra = 64.00 nm, which was higher than that of plain PLA membranes (Ra = 5.88 nm). These results were consistent with the literature, which suggests this is due to the high surface roughness value of the bone surface^77^.

Más et al*.* suggested that collagen had a net-like fibril structure So, a rise in the roughness of the surface after Col-I modification was expected^74^. The roughness of the Col-I modified BSM PLA membranes (Ra: 70.01 nm) was higher than that of unmodified BSM membranes (Ra: 50.02 nm), confirming the immobilization of collagen on the membranes.

Similarly, according to Bottino et al*.,* HA deposition on the surface led to a rise in the roughness^78^. Comparison of the roughness of the HA modified BSM PLA membranes with the unmodified membranes showed that HA modification increased the surface roughness to Ra = 71.87 nm. Although HA or Col-I modifications did not cause any deformation on the mimicked bone surface structure, these modifications increased surface roughness. Therefore, the results confirmed the mimicking quality and the change in surface features after Col-I immobilization and HA deposition on BSM PLA membranes.

### Culture of RAW 264.7 cells

#### Cell viability of RAW 264.7 cells

**Figure 5:**
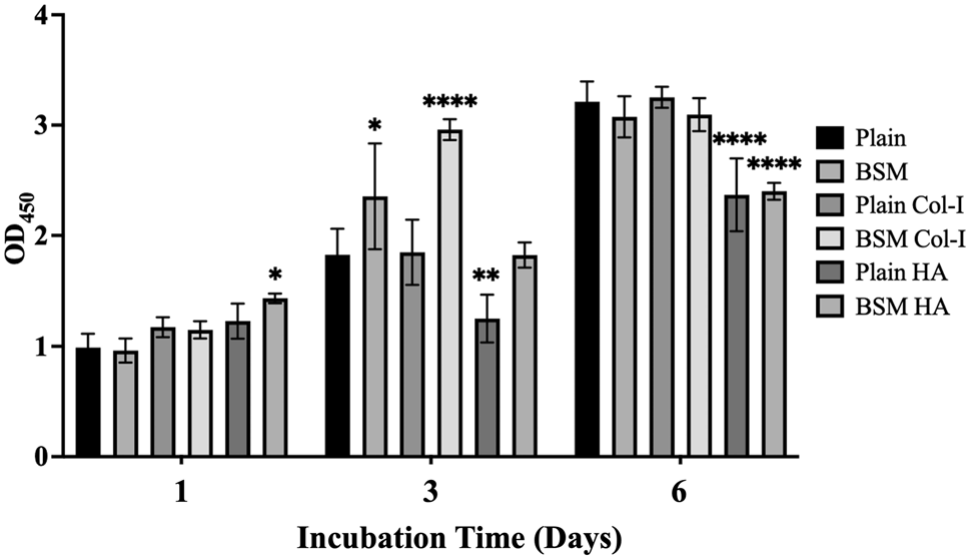
Viability of RAW264.7 cells adherent on unmodified, Col-I modified, and HA modified plain PLA and BSM PLA surfaces were measured at day 1, day 3, and day 6 (*p < 0.05, **p < 0.01, ***p < 0.001, ****p < 0.0001).

To investigate the impact of surface topography and chemical modifications on immune cells, we assessed the viability of RAW 264.7 cells on unmodified and modified plain and BSM membranes for 6 days (Fig. 5). The viability of cells was measured on day 1, day 3, and day 6. The results showed that surface chemical modifications of the membranes significantly influenced cell viability. HA modification on BSM PLA membranes significantly increased adherent cell viability compared to plain PLA membranes without HA modification (*p* < 0.01). Additionally, the Col-I modification of BSM membranes improved cell viability at day 3 (*p* < 0.0001) compared to the HA-coated plain and BSM membranes (OD 1.23 ± 0.13, 1.82 ± 0.09, respectively). Moreover, HA-coated plain and BSM membranes showed the lowest viability rate on day 3 (*p* < 0.0001) compared to the other groups.

The initial high viability observed on day 1 with HA- deposited BSM membranes suggests enhanced cell adhesion and spreading due to surface modification. Conversely, the sustained viability observed on day 3 with Col-I modified BSM membranes may be attributed to Col-I's role as a ligand for integrin receptors, facilitating integrin signaling pathways crucial for cell proliferation and differentiation^79,80^.

Previous studies have highlighted how surface chemical modifications influence immune cell behavior, affecting macrophage activation and inflammation. Ja et al*.* reported reduced M1 macrophage activation with DGEA, a collagen I-derived peptide, suggesting a potential mechanism for the improved viability observed with Col-I modification^81^, and Viji et al*.* demonstrated anti-inflammatory effects of a collagen sheet impregnated with neem extract^82^. Additionally, the observed lag in viability on the 3rd and 6th days for the HA-coated BSM membranes can be attributed to the differentiation of M0 macrophages into M1 or M2 phenotypes, which have different functions in the immune response. For example, Laquerriere et al*.* showed that small-diameter hydroxyapatite particles can activate immune cells to release cytokines that favor inflammation^15^, while Sadowska et al*.* found that hydroxyapatite-coated materials play a role in triggering the formation of pro-inflammatory cytokines and controlling osteogenic processes^34^.

Surface topography alone appears to have minimal initial impact on viability, as observed by Schaub et al. with PLA electrospun fibers, Majidi et al. with PDMS surfaces, and Wang et al. with smooth and rough titanium. In these studies, no significant differences in viability were noted at day 1, regardless of topography, after a day of incubation^83–85^. These findings suggest that initial cell adhesion and spreading may be less influenced by surface topography compared to surface chemistry. However, Wang et al*.* observed increased viability on rough surfaces by day 3, suggesting topographical influence over time^85^. This delayed effect might be due to changes in cell morphology, cytoskeletal organization, or mechano-transduction pathways triggered by surface features. Kim et al*.* reported no significant effect on cell morphology with collagen-incorporated PEG hydrogels, indicating collagen alone may not affect viability through topographical cues^86^. Li et al*.* observed no difference in viability at days 1 and 2 with Col-I modified samples, but noted changes in cytoskeleton and focal adhesion at day 3, suggesting delayed effects of surface properties on these aspects of cell behavior that could ultimately influence viability and macrophage polarization^87^. Finally, Neacsua et al. found that the addition of the M1 macrophage phenotype stimulator LPS resulted in decreased viability of macrophages on TiO_2_ nanotubes, while Schaub et al*.* reported similar findings with LPS-stimulated macrophages on PLA electrospun fibers^83,88^. Therefore, changes in viability observed on day 3 may be related to polarization, reflecting the complex interaction between surface properties, inflammatory stimuli, and cell behavior.

In summary, surface chemical modifications, such as HA or Col-I addition, significantly impact immune cell viability, with variations attributed to surface properties. While surface topography alone minimally affects viability initially, alterations in both topography and surface chemistry can synergistically enhance viability over time. These findings underscore the collective influence of surface topography, material composition, and macrophage polarization status on cell viability.

#### Analysis of cell morphology by scanning electron microscope (SEM)

**Figure 6:**
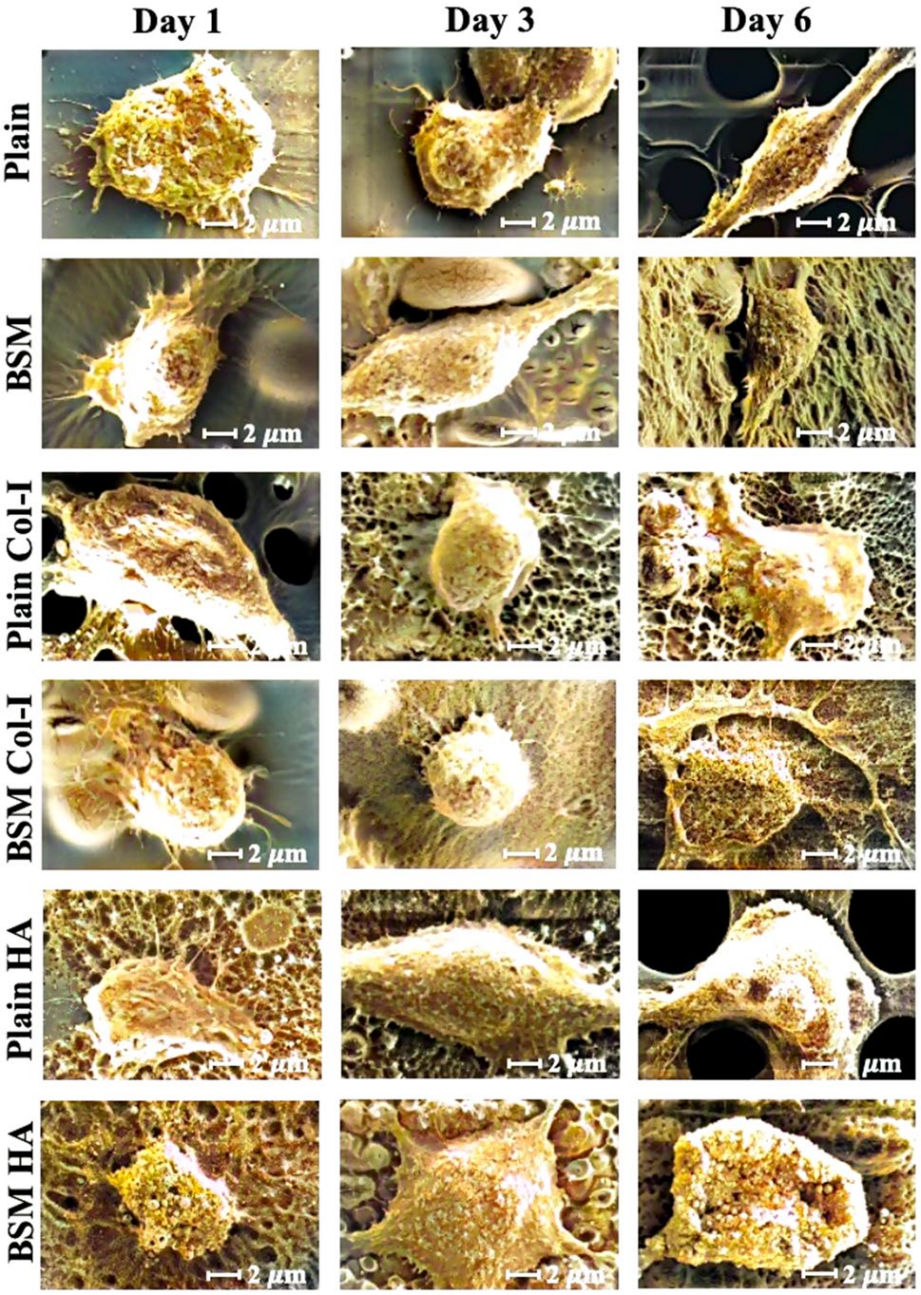
SEM images of unmodified, and Col-I and HA modified, plain and BSM membranes on day 1, day 3, and day 6 (Scale bars: 2 µm).

To investigate the effect of surface topography and Col-I and HA modifications on macrophage polarization, the morphology of RAW 264.7 cells on unmodified and modified plain and BSM membranes was examined on day 1, day 3, and day 6. The cells on the samples were observed by scanning electron microscopy (Zeiss EVO HD) and presented in Fig. 6.

According to Heinrich et al. M0-macrophages were predominantly observed as compact, circular cells, with an average diameter of around 8 μm. These cells displayed minimal to no cytoplasmic extensions, occasionally reaching a length of up to 1 μm^89^. On the first day, the cell morphology on each sample resembled that of M0 macrophages as described in existing literature. In all samples, cells were observed to be attached and displayed a small, rounded morphology with limited cytoplasmic extensions.

By day 3, if the produced membranes were osteoimmunomodulatory, the M1 macrophage phenotype was expected to be observed. The cells seeded on HA coated plain and BSM membranes showed an expanded amoeboid cell shape with rounded cell bodies and several fragile cytoplasmic extensions on the surface similar to the reported phenotype of M1 macrophages in the literature^89,90^.

The M2 macrophage phenotype comprises two cell types, the first being large "spindeloid" macrophages with an elongated cell body and cytoplasmic extensions at the apical ends of the cell bodies, and the second being giant multinucleated cells with plentiful cytoplasmic projections on the cellular surface^89,90^. By day 6, all cells on both unmodified and modified membranes exhibited a large "spindeloid" morphology, indicative of the M2 phenotype. Moreover, the M2 macrophage phenotype comprises four subtypes, each with distinct morphological features and functional roles: M2a, M2b, M2c, and M2d. M2a macrophages show elongated and narrowed morphologies. On the other hand, M2b macrophages are elongated and granular, whereas M2c macrophages are smaller and more circular. Finally, M2d macrophages are identified by their flattened and elongated morphology^91–93^. Macrophages on unmodified membranes resembled M2a macrophages, displaying an elongated and narrowed shape. In contrast, cells on modified membranes did not exhibit elongation and showed characteristics more akin to both M2b and M2c subtypes.

Consequently, sequential M1 and M2 macrophage polarizations were only observed in the cells adherent to HA-deposited plain and BSM PLA membranes. Our observations suggest that HA-deposited plain and BSM PLA membranes might promote initial M1 polarization at day 3, followed by a shift towards M2b or M2c polarization by day 6. However, conclusive evidence for sequential M1 and M2 polarization is limited to HA-deposited plain and BSM PLA membranes and requires further investigation.

#### Cytokine content analysis in the supernatant

**Figure 7:**
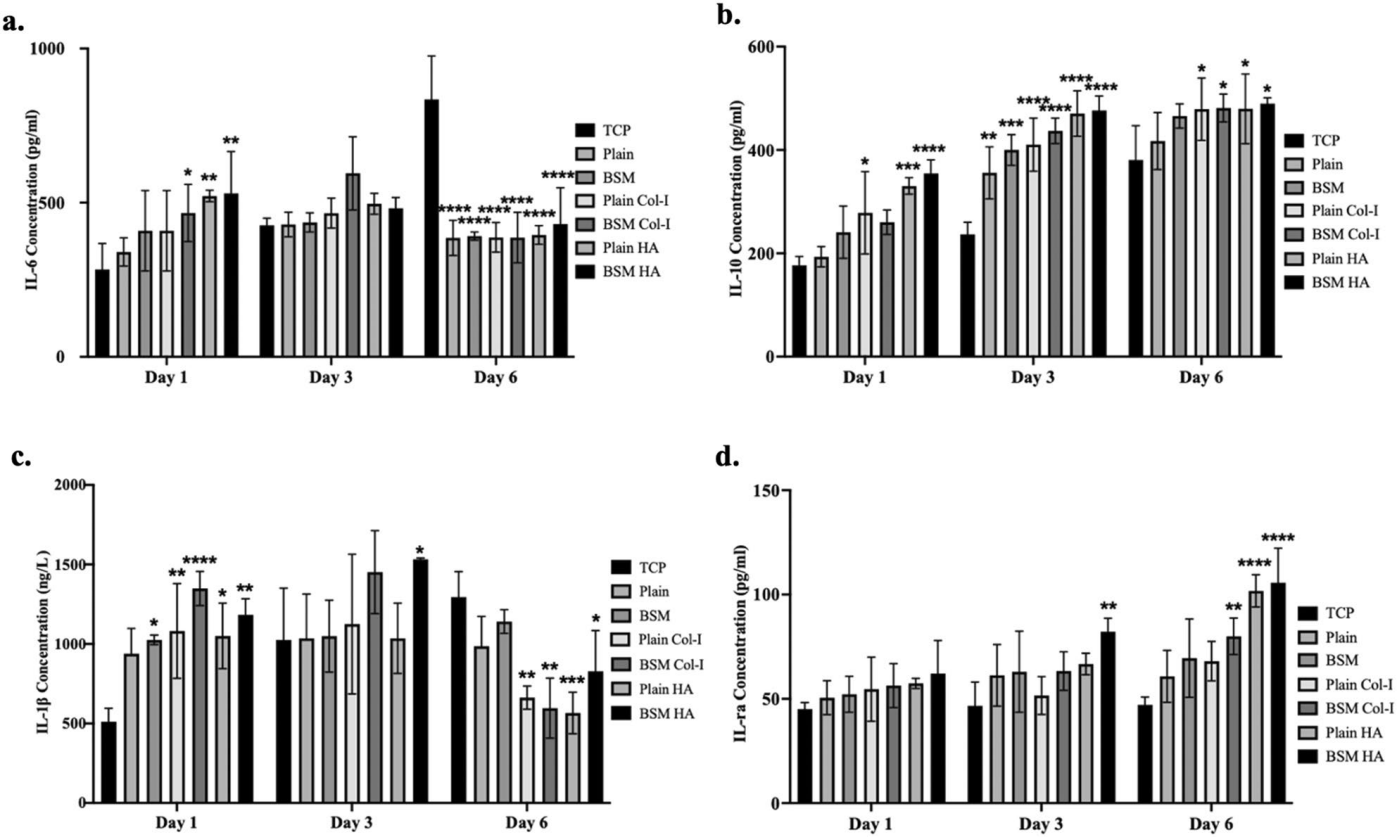
Pro-Inflammatory and Anti-Inflammatory factor content and amount analysis on unmodified and Col-I and HA modified, plain PLA and BSM PLA membranes by days. (**a**) IL-6 Concentration, (**b**) IL-10 Concentration, (**c**) IL-1β Concentration, d. IL-1ra Concentration (*p < 0.05, **p < 0.01, ***p < 0.001, ****p < 0.0001).

To understand the dominant macrophage phenotype in the regulation of osteoimmunomodulation, the ratio of proinflammatory and antiinflammatory factors of monocytes/macrophages was determined colorimetrically with double antibody sandwich ELISA kits. Cells seeded on unmodified and modified, plain PLA and BSM PLA membranes were incubated for 1 day, 3 days, 6 days, and the presence of proinflammatory (IL-6 and IL-1β) and antiinflammatory (IL-1ra and IL-10) factors in the medium and the amount of this cytokine were analyzed using ELISA^62,63^. For the analysis, first, a calibration curve was obtained to find the concentration of each cytokine. Concentration calculations were made for each cytokine and the results are presented in Fig. 7.

Wound healing challenges often arise from disruptions in transitioning from M1 to M2 macrophages. Biomaterials facilitating sequential M1-to-M2 macrophage activation are proposed to enhance healing^5–7,9^. Typically, M1 macrophages dominate early wound healing (days 1–3) before transitioning to M2 macrophages (days 4–7)^10^. Thus, it was expected that the highest levels of proinflammatory cytokines IL-6 and IL-1β would be observed on the 3rd day, followed by a decrease, while anti-inflammatory cytokines IL-1ra and IL-10 would steadily increase, peaking on the 6th day. This pattern would demonstrate the immunomodulatory effect of our biomaterial.

Regarding our observations, first, IL-6 cytokine, which is known as proinflammatory, was released in all samples the most on the 3rd day, and then the rate of release increased in control group (TCP) on the 6th day but decreased in all other samples. The IL-6 release rate of HA deposited samples was statistically different on days 1 and 6 compared to TCP. On the 6th day, the cytokine release of all our samples is statistically different from the cells seeded on the TCP. When it comes to the analysis of second proinflammatory, IL-1β cytokines, the amount of cytokine released from the HA- deposited BSM membranes was significantly statistically different from the adherent cells on the cell culture dish on all days. For IL1ra and IL-10, which are anti-inflammatory cytokines, the release increased day by day on all samples and this increase is consistent with the literature. For IL-10, the groups that were most statistically different from the cell culture dish on all days were HA and Col-I modified BSM membranes, while for IL-1ra, the group that was most statistically different from the cell culture dish when all days were considered was HA coated BSM membranes.

Despite differences in surface morphology between plain and BSM PLA surfaces, the Col-I and HA-modified BSM PLA surfaces exhibit hydrophilic properties. This distinction highlights a significant contrast: while plain and BSM PLA membranes are hydrophobic, the modifications make them hydrophilic. This indicates that the variations in immune responses observed among unmodified and modified samples are likely influenced more by changes in their surface chemistry and topography, which collectively affect surface wetting properties, rather than differences in surface topography alone. This perspective finds support in several studies demonstrating that alterations in surface chemistry, along with morphology, notably impact macrophage behavior and polarization. Neacsua et al. observed that under standard culture conditions, surface topography alone did not significantly influence macrophage inflammatory activity by investigating the interactions between titania nanotubes and commercial pure titanium with macrophages under standard and pro-inflammatory conditions^88^. Hotchkiss et al*.* conducted experiments with macrophages cultured on various titanium surfaces with different morphologies and wettability. They found that surface wettability influences macrophage polarization, with hydrophilic rough surfaces inducing an anti-inflammatory M2-like state, while smooth hydrophobic surfaces promoted inflammatory M1-like activation after 3 days of incubation^94^. Similarly, Lv et al*.* demonstrated that hydrophilic TiO_2_ surfaces exhibit enhanced anti-inflammatory and pro-healing performance of macrophages compared to hydrophobic ones^95^. Additionally, Gao et al*.* produced super-hydrophilic TNT surfaces by hydrogenation, which did not cause a change in nanotubular topography or introduce impurities. They observed that the super-hydrophilic nanotubular surface preferentially activated macrophages toward an anti-inflammatory phenotype under standard conditions^96^. Furthermore, our observations indicate that HA-deposited samples, being the most hydrophilic, demonstrated M1 polarization on day 3 and M2 polarization on day 6. This suggests a dynamic interaction between surface chemistry, morphology, and immune response over time. Our results align with existing literature, suggesting that HA-deposited BSM samples, considering their SEM images, appear to be the most osteoimmunomodulatory samples. Thus, in our study, the regulation of immune responses is primarily driven by the synergistic effect of modifications and surface morphology due to surface hydrophilicity.

To gain a deeper understanding and achieve more precise classification of the M1/M2 phenotypes, future studies should incorporate additional techniques such as flow cytometry and RT-PCR to evaluate the effect of HA-deposited BSM membranes on osteoimmunomodulation. However, our results show potential advantages of bone surface mimic biomaterials on osteoimmunomodulation.

## Conclusion

Our study employed a biomimetic approach to replicate bone surface topography and chemical structure, resulting in the production of osteoimmunomodulative biomaterials which promote wound healing. We have demonstrated the crucial role of bone surface topography in regulating the immune response and highlighted the need to develop biomaterials that can induce “synergistic and sequential” M1 and M2 macrophage polarizations. Our results show that HA-coated BSM PLA membranes have exhibited the best immunomodulatory effect between all experimental groups, such as unmodified and Col-I modified plain and BSM PLA membranes as demonstrated by sequential M1 and M2 polarization of RAW 264.7 cells cultured on them. Physical, chemical, and biochemical properties of biomaterials play a vital role in determining the dominant macrophage phenotype and immune response. This study highlights the potential of biomaterials that mimic bone surface topography to modulate the osteoimmune response and may help lay the groundwork for future research in the biomaterials field. While our initial stages focused on establishing a foundational proof of concept, we recognize the necessity of methodological optimization. Future studies should contain additional techniques such as flow cytometry and RT-PCR to observe the effect of HA-coated BSM membranes on osteoimmunomodulation, enhancing our understanding and enabling more accurate M1/M2 phenotype and M2 subtype classification. Furthermore, it is crucial to explore the versatility of our technique and its prospective application to different bone tissue models. By extending our approach to various bone tissue types, we aim to gain a comprehensive understanding of how diverse surface topographies influence cell behavior, contributing to a more insightful perspective in biomaterials research.

### Supplementary Information

Supplementary Figures.

## Data Availability

The authors declare that the data supporting the findings of this study are available within the paper and its Supplementary Information files. Should any raw data files be needed in another format they are available from the corresponding author upon reasonable request.
