# Supplementary material for: Effects of bone surface topography and chemistry on macrophage polarization
Source: Sci Rep. 2024 Jun 3;14:12721. doi: 10.1038/s41598-024-62484-3 (PMC11148019; doi:10.1038/s41598-024-62484-3)
Supplement: Supplementary file 1 — Supplementary Figures. [file 41598_2024_62484_MOESM1_ESM.docx]

**THE EFFECT OF BONE SURFACE TOPOGRAPHY ON MACROPHAGE POLARIZATION**

Birgün Özçolak^a,b^, Berkay Erenay^a^, Sedat Odabaş ^c,d^, Klaus D. Jandt^e^ and Bora Garipcan^a^*

^a^Biomimetics and Bioinspired Biomaterials Research Laboratory, Institute of Biomedical Engineering, Boğaziçi University, Istanbul, Turkey

^b^Department of Biomedical Engineering, School of Engineering and Natural Sciences, Istanbul Medipol University, Istanbul, Turkey

**^c^**Biomaterials and Tissue Engineering Laboratory (bteLAB), Faculty of Science, Department of Chemistry, Ankara University, 06560, Turkey

^d^Interdisciplinary Research Unit for Advanced Materials (INTRAM), Ankara University, Ankara, 06560, Turkey

^e^Chair of Materials Science (CMS), Otto Schott Institute of Materials Research, Faculty of Physics and Astronomy, Friedrich Schiller University Jena, Löbdergraben 32, 07743 Jena, Germany


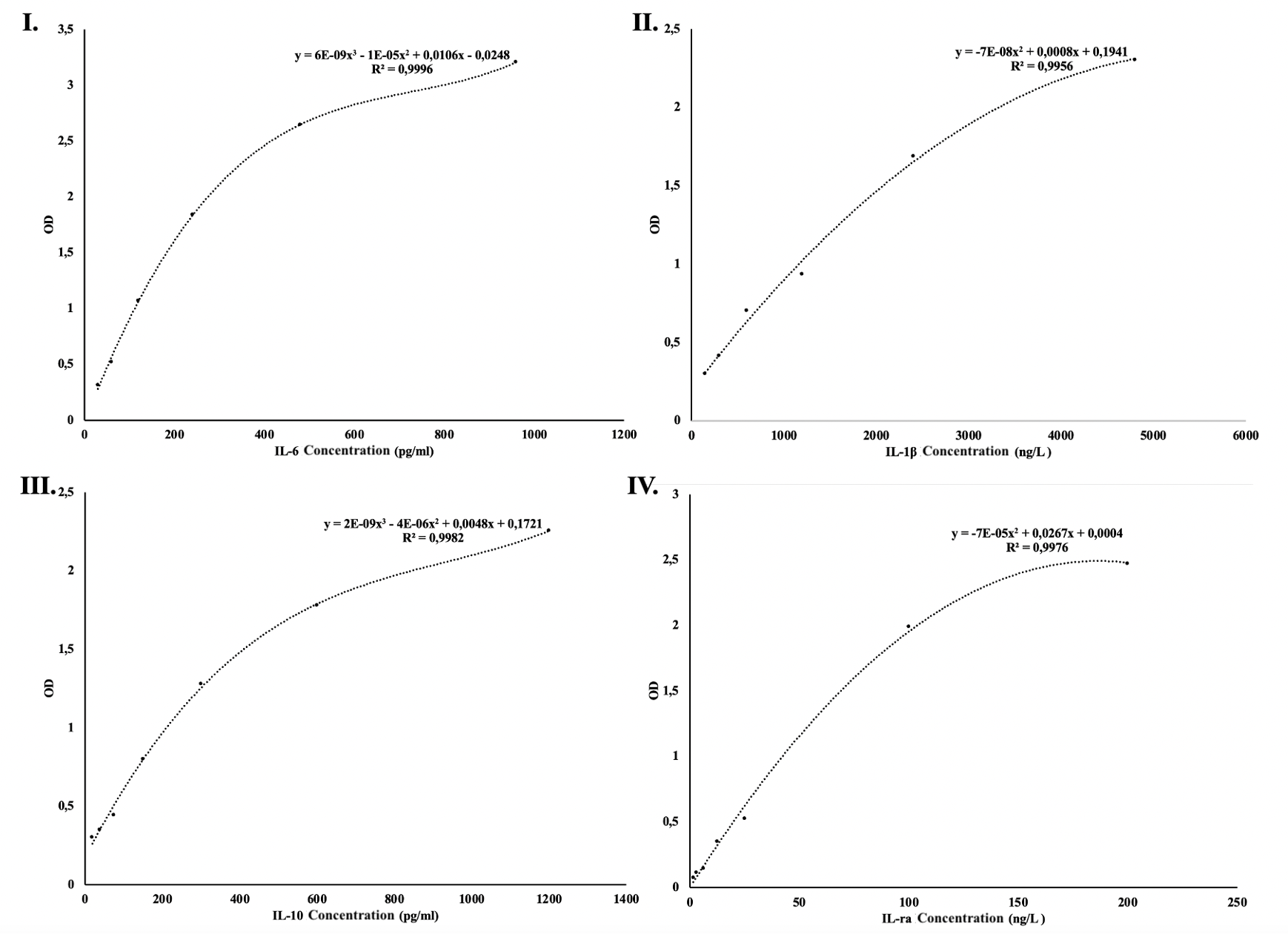


**Figure S1:** Calibration Curves. I. IL-6 Calibration Curve, II. IL-1β Calibration Curve, III. IL-10 Calibration Curve, IV. IL-1ra Calibration Curve.


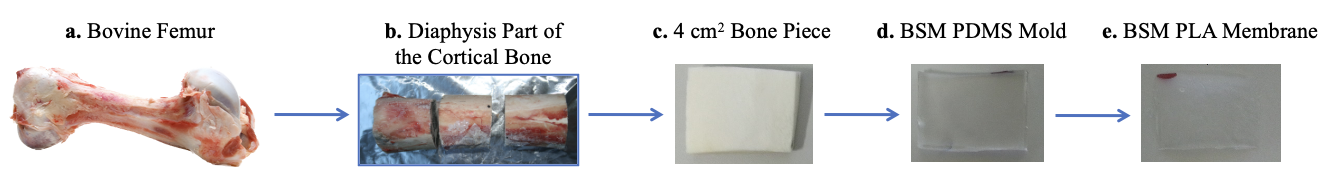


**Figure S2:** Schematic Overview of BSM PLA Membrane Production: a. Procurement of Bovine Femur from a Local Butcher, b. Extraction of Diaphysis Cortical Bone, c. Obtained Clean Bone Pieces (2 cm x 2 cm), d. BSM PDMS Mold Production, e. BSM PLA Membrane Production.
